# Adolescent food insecurity in rural Sindh, Pakistan: a cross-sectional survey

**DOI:** 10.1186/s40795-020-00343-w

**Published:** 2020-03-26

**Authors:** Sana Sheikh, Romaina Iqbal, Rahat Qureshi, Iqbal Azam, Rubina Barolia

**Affiliations:** 1 Department of Obstetrics and Gynecology, Private wing 2nd floor, Aga Khan University, Stadium road, Karachi, 74800 Pakistan; 2 Department of Community Health Sciences, Ibn-e-Ridwan building, Aga Khan University, Stadium road, Karachi, 74800 Pakistan; 3 School of Nursing and Midwifery, Aga Khan University, Stadium road, Karachi, 74800 Pakistan

**Keywords:** Adolescents, Food insecurity, Lower-middle-income, Rural, Pakistan

## Abstract

**Background:**

Food insecurity (FI) is alarmingly high in developing countries including Pakistan. A quarter of Pakistan’s population consists of adolescents yet there is no information on their experience of FI. FI at adolescent age have long term effect on mental and physical health hence we aimed to determine the prevalence of food insecurity (FI) among adolescents and compare it with household FI, and assess social determinants of adolescent FI.

**Methods:**

A cross-sectional survey on 799 households with unmarried adolescents was conducted from September 2015 to June 2016 in three union councils of Hyderabad, Pakistan. Unmarried 10–19 years old girls and boys were interviewed regarding their FI status using Household Food Insecurity Assessment Scale (HFIAS). Household-level FI was also assessed by interviewing mothers of adolescents, and it was compared with adolescent’s FI. Association of adolescent’s FI with socio-demographic determinants was explored through Cox regression using STATA version 14.0. and prevalence ratios were estimated.

**Results:**

FI was found among 52.4% of the adolescents compared to 39% of the households. Thirty percent of the adolescents were food insecure within the food secure households. Female adolescents were found to be less food insecure (Adjusted Prevalence Ratio (APR) 0.4 95% CI [0.3, 0.5]) compared to males. Social determinants like socioeconomic status (SES), crowding index or education of parents were not associated with adolescents’ FI.

**Conclusion:**

Half of the adolescents were found to be food insecure which raises concerns regarding their health in the long run. Gender is an important social determinant of FI among adolescents which suggests an in-depth exploration of social dynamics of adolescent FI. We recommend the mixed-methods study to develop contextually relevant interventions to reduce FI among this group and improve their health status.

## Background

Every ninth person in the world is suffering from food insecurity (FI) [1]. Food insecurity is defined as a compromise on quantity or quality of food acquisition and consumption due to the lack of resources that hampers normal growth, development, and maintenance of a healthy life [2]. In adolescence, there is an increased demand for nutrients due to growth spurt and to attain puberty [3]. Studies have reported that food insecurity at adolescent stage (10–19 years of age) can affect their linear growth [4], high risk of involving in violence perpetration [5] and are twice likely to develop cardiovascular diseases (2.27; 95% confidence interval [CI], 1.61–3.21) [6].

Pakistan has been categorized among high-risk countries for FI by the Global Food Security Index (GFSI) 2019 [7]. On average 48% of Pakistan’s population is food insecure and with comparatively high FI in rural areas (60.6% rural vs. 52.4% urban) [8]. Current literature on FI from Pakistan and other south Asian countries is focused on women and children under 5 years of age [9–12]. Data on effects of FI on adolescents is scarce globally compared to data on children. A comparative study in United States (US) found higher odds of mental health impairment among adolescents (OR: 1.33 [95% CI: 1.05, 1.68]) compared to children (OR: 1.26 [95% CI: 1.05, 1.52]) in severely food insecure households [13]. Another study reported adolescent diet being suffered most due to FI compared to younger age children [14].

Adolescents make 24% of Pakistan’s population but there are no data on FI estimates of adolescents and its socio-demographic factors [15]. Literature reports that 16–17% adolescents are food insecure in US and Canada [16, 17] whereas in developing countries like Ethiopia, Lebanon, and Venezuela 50% of the adolescents are found food insecure [18–20]. Belchew et al. found the place of residence, lower socio-economic status, dependency ratio, household FI and gender as social determinants of FI among Ethiopian adolescents [18]. Similar factors were also reported from Canada and Brazil [17, 21].

Many studies captured FI among adolescents by indirect response from their caregivers. Globally different studies have determined discordance between reporting of FI by parents and children [19, 22–25]. Consequently aim of this study is to determine the prevalence of food insecurity among adolescent when they are respondent for themselves and compare it with the household FI. It is postulated that during the food crisis adults try to protect the younger ones by cutting down the variety and quantity of their own food and buffer the effect of FI [26, 27]. Hence we expect that adolescents experience less FI compared to their households. FI at the household level and its comparison with adolescent’s individual FI has been studied in different settings and found that adolescents may not be protected from FI despite the efforts of the adults in the household [18, 19, 28]. Hence we aim to 1) determine and compare prevalence of FI among adolescents with the household-level FI 2) assess social determinants of FI among adolescents in rural Sindh.

## Methods

A cross-sectional survey was conducted from September 2015 to June 2016 where data was collected over 5 months (October 2015 to February 2016) in three union councils of Hyderabad, Pakistan. Hyderabad is the second largest district in Sindh province. Out of five provinces of Pakistan, the FI situation is worst in Sindh province where FI ranges from 40 to 70% [29]. Hyderabad has a population of 4.5 million with 40% rural population. It is further divided into four sub-districts, Hyderabad city, Hyderabad rural, Latifabad, and Qasimabad. We conducted this study in three union councils of sub-district Hyderabad rural namely Masubhurgari, Tando Hyder and Khatyan [30]. The average population of one union council is 30,000. Common language in the area is Urdu and Sindhi, and 90% of the residents are Muslims. Less than half of the females (43%) and 67% of the males are literate. Predominant occupation is agriculture in this sub-district and in the studied union councils [31].

**Fig. 1:**
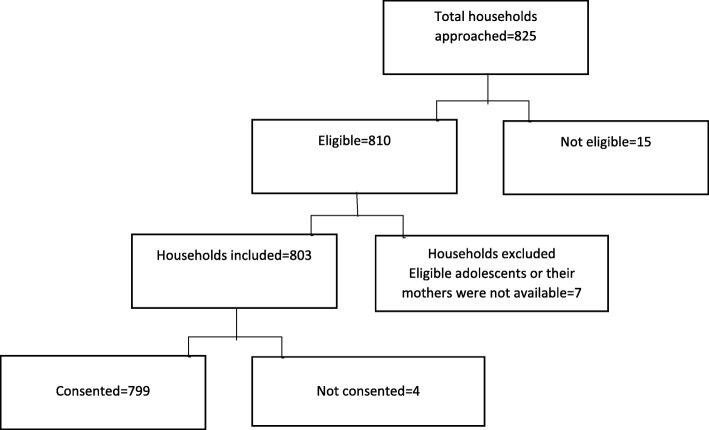
Participant flow diagram

Households with unmarried adolescents i.e. 10–19 years old girls and boys [32] living in the study area for the past six months participated in the study. Boys and girls with self-reported known co-morbidities which could affect their diet intakes such as chronic renal/cardiac disease, cancers of all types, thalassemia major or other blood disorders and diabetes were excluded. We recruited 799 participants through non-probabilistic sampling (Fig. 1). The research team visited house to house and looked for an eligible adolescent. If any household had more than one eligible participant, then the name of all eligible participants was written on a piece of paper and then the participant was selected through a lucky draw. Study purpose and procedures were explained to the adolescents and their mothers and they were asked for written the (< 18 years) or consent as appropriate. Written consent was also taken from parents of participants less than 18 years of age. If the participant was not literate then consent and assent were read out to them by a research team member in the presence of one witness and thumb impression of participant and witness was taken on consent /assent form. Literate participants signed the consent/assent form. No information was collected from the participants who refused the consent. Mothers and adolescents were interviewed separately for FI assessment in privacy to avoid any influence on answers by the presence of the family members.

Data on household-level socio-demographic factors like household assets, age and education of mother and father was collected through a structured questionnaire. Mother was the respondent for socio-demographic factors and a separate written informed consent for household-level data was obtained from her. Information related to FI was collected using the Household Food Insecurity Access Scale (HFIAS). This tool was developed by USAID’s Food and Nutrition Technical Assistance (FANTA) group in early 2000 [33] and it has been validated in Pakistan and other LMICs [20, 34]. HFIAS has 9 core questions regarding food intake occurring over the last four weeks (30 days). For every question frequency of occurrence is assessed (rarely, sometimes, often). Core questions capture the range of food insecurity from the anxiety of running out of food, compromise on quality or variety of diet, cutting down the portion size and disrupted eating patterns and sleeping hungry. Separate questionnaires were administered to mothers and adolescents for FI assessment. Mothers responded to household-level FI. HFIAS questions were rephrased to capture individual-level food insecurity among adolescents. Additional file 1: Adolescent and household questionnaire for FI.

Following the instruction of HFIAS four ordinal categories of food insecurity were developed (food secure, mild insecure, moderate insecure and severe food insecure). The food secure category was assigned when responses to all the nine items were no or anxiety over food was experienced rarely. The mild food insecure category corresponds to worrying sometimes or often for food, and/or eats a limited variety, and/or have undesirable food. Moderate category means a monotonous diet, and/or undesirable food is eaten and portion size is reduced sometimes. Reducing portion size often, and/or skipping meals, and/or going hungry even rarely qualifies for severe food insecurity [33]. All the responses given are scored. A minimum score of zero indicated the most food-secure households/individual, and a maximum score of 27 indicating the most food-insecure households or individuals. These categories were later collapsed to make a binary variable i.e. food-secure and food-insecure for regression analysis. The food-insecure category was made by combining mild, moderate and severe food-insecure categories.

Sample size estimation: This study has multiple objectives and the study sample size was calculated for all the objectives. But the sample size for the prevalence of stunting in adolescents came out largest and was used. The sample size was calculated using the WHO sample size calculator keeping prevalence of stunting 47%, [35] anticipated difference 5%, the sample size of 783. In this paper, we are focusing on the objective of FI among adolescents. Other objectives will be addressed in future papers.

Statistical analysis: Data was analyzed using Stata version 15.0. For descriptive statistics means ± standard deviations and proportions were calculated for continuous and categorical variables respectively. Based on descriptive statistics, categories of mother and father education were made. Father education was divided into no education, less than 10 years and more than 10 years of formal education. For mothers, the same education categories were not possible and data distribution depicted only two possible categories i.e. illiterate and literate.

Three categories of SES were developed based on the wealth index. Wealth index was calculated using possession of household items like animal cart, refrigerator, and motorbike, etc. First scores were assigned to household items by estimating the number of individuals having the item and subtracting it from the total sample size. The resulting number was assigned a score of the item. This way the item which was most common got the least score. After this, the score of all items possessed by an individual was summed up and a continuous wealth index was developed. The continuous index was divided into tertiles [36]. To make results interpretable the first tertile was labeled as low socio-economic, second as middle and last as high SES.

Inferential statistics were estimated by univariate analysis applying student’s t-test and chi-square and multivariable analysis using Cox regression after checking assumptions. Cox regression is a recommended statistical analysis to estimate prevalence ratios which is an appropriate measure in cross-sectional studies [37]. Crude and adjusted prevalence ratios (APR) were calculated with a 95% confidence interval. Multivariable analysis was adjusted for the age of the adolescent, sex, number of siblings, mother and father’s education and SES. Interactions between household FI and father’s education, household FI and mother’s education, father’s education and SES and father’s and mother’s education were checked, and none were significant at a *p*-value of 0.20.

## Results

Altogether, 799 adolescents, were recruited in the study out of which 399 (49.9%) were males. The mean age of adolescents was 13.5 years (SD 2.7 years). About a quarter (*n* = 217 (27.2%)) never attended school and 38.6% (*n* = 309) has 5 years of schooling. The total number of siblings ranges from zero to 14 with a mean of 5.3 (SD 2.2) siblings per participant. Only 15.5% (*n* = 124) of mothers had the education of one or more years compared to 53.9% of the fathers (Table 1).

**Table 1:** Baseline characteristics of adolescents

Variables	Total ***n*** = 799 n (%) Mean ± SD
Gender:	
Males	399 (49.9)
Females	400 (51.1)
Age (years)	13.5 ± 2.7
Years of schooling	3.7 ± 3.5
Number of siblings	5.3 ± 2.2
Number of adolescent siblings	2.2 ± 1.5
Male siblings attending school	1.2 ± 1.3
Female siblings attending school	0.63 ± 0.93
Father’s years of schooling	4.8 ± 5.15
Mother’s years of schooling	0.96 ± 2.5
Socio-economic status:	
Lower	298 (37.3)
Middle	255 (31.9)
High	246 (30.8)

**Fig. 2:**
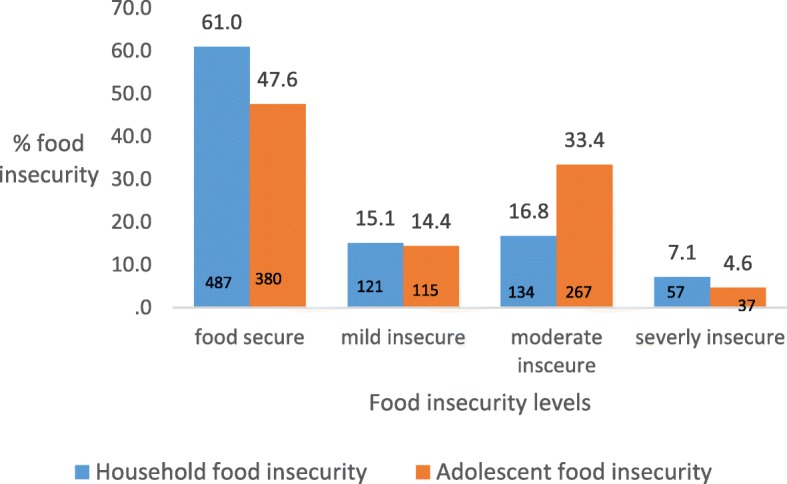
Distribution of household (*n* = 799) and adolescent (*n* = 799) food insecurity status

There were 4.6% (*n* = 37) of the adolescents who reported experiencing severe FI whereas 7.1% (*n* = 57) of the households were severely food insecure. Half of the adolescents (47.6%, (*n* = 380)) and 61% (*n* = 487) of the households were food secure (Fig. 2).

For inferential analysis, 11 cases were excluded because of missing information for socio-demographic variables and data of 788 participants were used. No data were missing for FI status. For regression, the binary variable of food-secure and food-insecure was used. Half of the adolescents were found to be food-insecure (52.6%) compared to 39% of the households.

In food secure households, 65.2% of boys were food insecure compared to 3% of girls. Food insecure adolescents in food-secure households were 1.2 ± 0.1 years younger and fewsmothers were illiterate (13.4% vs. 21.4%) in this group compared to food secure adolescents (Table 2).

**Table 2:** Comparison of characteristics of food secure and insecure adolescents in food-secure households

Variables	Food secure adolescents ***N*** = 337 N (%) Mean ± SD	Food insecure adolescents ***N*** = 142 N (%) Mean ± SD	***P***-value
Age of the adolescent	14.0 ± 2.7	13.2 ± 2.8	< 0.01
Father’s years of schooling			
No schooling	143 (42.4)	54 (38.0)	0.21
< 10 years	70 (20.8)	40 (28.2)	
≥ 10 years	124 (36.8)	48 (33.8)	
Mother’s years of schooling			
Illiterate	265 (78.6)	123 (86.6)	0.04
Literate	72 (21.4)	19 (13.4)	
Wealth tertiles			
Low	77 (22.8)	42 (29.6)	0.29
Middle	101 (30.0)	40 (28.2)	
high	159 (47.2)	60 (42.3)	
Total number of siblings	5.1 ± 2.3	5.3 ± 2.1	0.43
Gender			< 0.01
Male	72 (21.4)	132 (93.0)	
Female	265 (78.6)	10 (7.0)	

Chi-square test was used to compare the characteristics

Among the adolescents living in food-insecure households, 92% of the boys and 82% of the girls were food insecure. Only 8% of mothers were literate in food insecure group compared to 19% of literate mothers in food-secure adolescents (Table 3).

**Table 3:** Comparison of characteristics of food secure and insecure adolescents in food-insecure households

Variables	Food secure adolescents ***N*** = 36 N (%) Mean ± SD	Food insecure adolescents ***N*** = 273 N (%) Mean ± SD	***P***-value
Age of the adolescent	13.5 ± 2.5	13.1 ± 2.4	0.37
Father’s years of schooling			
No schooling	21 (58.3)	143 (52.4)	0.62
< 10 years	6 (16.7)	65 (23.8)	
≥ 10 years	9 (25.0)	65 (23.8)	
Mother’s years of schooling			
Illiterate	29 (80.6)	250 (91.6)	0.03
Literate	7 (19.4)	23 (8.4)	
Wealth tertiles			
Low	13 (36.1)	133 (48.7)	0.11
Middle	16 (44.4)	106 (38.8)	
high	7 (19.4)	34 (12.5)	
Total number of siblings	5.2 ± 2.2	5.6 ± 2.3	0.37
Gender			
Male	14 (38.9)	179 (65.6)	< 0.01
Female	22 (61.1)	94 (34.4)	

Chi-square test was used to compare the characteristics

Household FI was significantly associated with adolescents’ FI and the prevalence of FI among adolescents was 2.3 times higher (95% CI 1.9, 2.9) if they belong to a food-insecure household compared to an adolescent who was in the food-secure household. Gender was also a significant contributor to adolescents’ FI. FI was 60% less prevalent if an adolescent was female in comparison to males (APR 0.4 [95% CI 0.3, 0.5]). Age of adolescents, education status of father or mother, size of the household and SES were not significant factors of adolescents’ FI in this study Table 4. None of the interactions were significant. Additional file 1.

**Table 4:** Factors associated with food insecurity among adolescents

Variables	Total adolescents ***N*** = 788 n (%) Mean ± SD	Food insecure adolescents ***N*** = 415 n (%) Mean ± SD	Crude Prevalence Ratio (95% CI)	Adjusted Prevalence Ratio (95% CI)
Food secure household				
Food insecure	479 (60.7)	142 (34.3)	1	1
household	309 (39.2)	273 (65.7)	2.9 (2.4, 3.6)	2.3 (1.9, 2.9)
Male	397 (50.3)	311 (74.9)	1	1
Female	391 (49.6)	104 (25.1)	0.3 (0.2, 0.4)	0.4 (0.3, 0.5)
Age of adolescent	13.54 ± 2·71	13.15 ± 2.60	0.94 (0.91, 0.98)	0.9 (0.9, 1.0)
Father’s years of schooling				
No schooling	361 (45.8)	197 (47.4)	1	1
< 10 years	181 (23.0)	105 (25.3)	1.0 (0.8, 1.3)	1.1 (0.8, 1.4)
≥ 10 years	246 (31.2)	113 (27.2)	0.8 (0.6, 1.0)	1.0 (0.8, 1.3)
Mother’s years of schooling				
Illiterate	667 (84.6)	373 (89.8)	1	1
Literate	121 (15.4)	42 (10.1)	0.6 (0.4, 0.8)	0.8 (0.6, 1.2)
Total number of siblings	5.35 ± 2.29	0 ± 5.51	1.0 (0.9, 1.0)	1.0 (0.9, 1.0)
Socio-economic status:				
Low	296 (37.6)	188 (45.3)	1.8 (1.4, 2.3)	1.1 (0.8, 1.5)
Middle	251 (31.8)	143 (34.4)	1.6 (1.2, 2.1)	1.1 (0.8, 1.5)
High	241 (30.5)	84 (20.2)	1	1

Model adjusted for the age of the adolescent, sex, number of siblings, mother and father’s education and SES

## Discussion

We observed that FI is higher among adolescents (52%) compared to households (39%), gender is significantly associated with food insecurity of adolescents and it is more prevalent among boys. Household FI is associated with adolescent FI even after adjusting for other socio-demographic factors.

Generally, it is assumed that household-level FI reflects individual FI, but studies have shown that it might not be true [22, 23]. We also found FI among 68% of the adolescent living in food-secure households. This emphasizes that the measurement of household-level FI may not be a true reflection of adolescents FI and should be measured separately. Adolescents are at an age where they can sense the stress in the house and try to play their part in buffering the effects of FI [25]. Literature reports that adolescents voluntarily cut-down their portion size during food shortages which at times go unnoticed by adults of the family [38]. Frongillo explained a few reasons for parents’ lack of information regarding adolescent FI through their qualitative work. The author wrote that when parents reduce their portion sizes, do not discuss the FI situation of households with their children, they assume that they have protected the children from hunger and anxiety of FI [25]. Hence, adolescents’ FI is under-reported by adults of the household. However, adolescents take cue from the change in household meals or expenditure pattern and are aware of FI in household. They give up their share of food or generate resources to improve the FI situation [25, 39, 40].

While assessing the social determinants of adolescent’s FI study found household FI to be an independent determinant. The same has been reported in studies conducted in Ethiopia (Adjusted odds ratio (AOR) = 2.86, *P* < 0.001) [18]. It can happen when households experience FI for a longer duration then adults cannot buffer its effects and young members are exposed to FI [18]. Our study was for a short duration and cannot differentiate between acute and chronic FI among participants. Other than household FI, none of the variables at the household level are associated with adolescent FI in this study. The number of siblings served as the proxy indicator for household size in the study and it has no association with adolescent FI. This study’s finding is consistent with existing literature where household size is not a significant factor of adolescent’s FI [18, 26]. We did not find an association between SES of households and FI for rural adolescents. SES has been documented as a significant factor of FI by other studies (low-income households, AOR = 1.61, *P* < 0.026) [17, 18]. However, literature has inconsistent findings on the effect of household income on FI. Our findings support the perception that poverty is not responsible alone for FI rather requires a multilevel exploration of other determinants. These determinants include community neighborhood (access to food retail store), social support, tobacco or substance use by family members, food price fluctuations, unexpected events like medical or other expenses, etc. [41].

In our study FI is found more among boys compared to girls. The same pattern was reported from Bangladesh where it was observed that in food insecure household men were more likely to consume less food [42]. Belchew et al. reported biased food distribution within the household among male and female adolescents in Ethiopia resulting in high FI (AOR 1.46, 95% CI 1.12, 1.92) among girls [43]. A systematic review stated mixed results for the role of gender on intra-household food allocation in South Asia [44]. National Nutrition Survey (NNS) 2011 of Pakistan captured mothers’ responses on gender discrimination in food distribution among children and found no discrimination by them [8]. The inconsistent results reflect the complexity of the FI phenomenon and a need to explore what is being perceived by FI. Understanding of food deprivation is shaped by culture and societal norms and can be contextually driven. Because of patriarchal society females in South Asia are habitual to demand less food than their body requirement [45] . This could have affected the reporting of FI amongst young females for themselves.

We also observed that girls’ response to FI was less discordant to household FI compared to boys (8% for girls vs. 37% for boys). The higher concordance of FI response between households and females has also been observed in Burkina Faso among adult women. Nanama et al. found that households with multiple wives, the youngest wife experience similar FI as of the household. Authors posited that this occurs because of the less autonomy and higher dependency of young wives on their households that tie the increase and decrease of their FI with that of the households compared to older wives [46]. We anticipate that patriarchal culture in Pakistan allows male adolescents to bargain for better food and express their voice if they are not being provided with the food of their choice regardless of the situation of the household FI. Hence, their FI can be different from household FI. Whereas, females are more conditioned to accept and go along with the household situation and hence have similar FI experience as that of the household.

The strengths of our study include rural community-based data of adolescents and the estimation of their FI by responses from themselves and it has an adequate sample size to answer the multiple research questions. The literature on adolescent FI is relatively scarce from south Asia and this study is a contribution to the existing literature on adolescent FI from this part of the world.

It was a cross-sectional survey hence causality of socio-demographic factors with adolescents’ FI cannot be established. However, it is advisable to conduct a survey first if baseline data is not available on the study topic, so the design of the study was justified. One limitation of the study was the non-availability of data on the employment status of adolescents and water sanitation and hygiene (WASH) indicators. Both of these factors are important for the effective absorption of the nutrients and can affect individual-level FI. In our opinion, findings of our study may have not been affected as the purpose was to elicit the information on acquisition of food and issue of absorption of nutrients comes later. Another limitation is non-probability sampling which can limit the generalizability of the results. But the communities we studied are homogenous socially, culturally and have a similar lifestyle. Hence we think results are generalizable to the communities with similar contexts. We did not account for the clustering effect hence it is advised to interpret statistical significance with caution. Though with a few numbers of clusters it is unlikely that clustering affects the estimates [47].

## Conclusion

We found that FI is prevalent among adolescents, more in boys than girls and half of the adolescents are food insecure. This warrants immediate attention by the government as the measurement of FI at the household level may mask FI at the individual level; especially in the adolescent age group. We feel that an in-depth investigation of the socio-cultural dynamics of FI is needed. We recommend mixed-methods studies to unpack the complexities around this phenomenon and then design contextually relevant interventions to reduce FI among this group and improve their health status.

## Supplementary information

**Additional file 1.**

## Data Availability

The datasets used and/or analyzed during the current study are available from the corresponding author on reasonable request.

## Abbreviations

APR: Adjusted prevalence ratio
AOR: Adjusted odds ratio
CI: confidence interval
FI: Food insecurity
HFIAS: Household Food Insecurity Access Scale
LMIC: Lower-middle-income countries
NNS: National Nutrition Survey
OR: Odds ratio
SD: Standard deviation
SES: Socioeconomic status
WASH: Water, sanitation, and hygiene
