# Supplementary material for: Adolescent food insecurity in rural Sindh, Pakistan: a cross-sectional survey
Source: BMC Nutr. 2020 Mar 26;6:17. doi: 10.1186/s40795-020-00343-w (PMC7098077; doi:10.1186/s40795-020-00343-w)
Supplement: Supplementary file 1 — Additional file 1. [file 40795_2020_343_MOESM1_ESM.docx]

**Appendix A**

**Adolescent questionnaire for assessment of food insecurity**

| S.No | Questions | Responses |
| --- | --- | --- |
|  | In the past four weeks, did you worry that your household would not have enough food? | 0 = No (skip to Q2)  1=Yes  ….\|___\| |
| 1.a. | How often did this happen? | 1 = Rarely (once or twice in the past four weeks)  2 = Sometimes (three to ten times in the past  four weeks)  3 = Often (more than ten times in the past four  weeks)  ….\|___\| |
|  | In the past four weeks, were you not able to eat the kinds of foods you preferred because of a lack of resources? | 0 = No (skip to Q3)  1=Yes ….\|___\| |
| 2.a. | How often did this happen? | 1 = Rarely (once or twice in the past four  weeks)  2 = Sometimes (three to ten times in the past  four weeks)  3 = Often (more than ten times in the past four  weeks)  ….\|___\| |
|  | In the past four weeks, did you have to eat a limited variety of foods due to a lack of resources? | 0 = No (skip to Q4)  1 = Yes  ….\|___\| |
| 3.a. | How often did this happen? | 1 = Rarely (once or twice in the past four  weeks)  2 = Sometimes (three to ten times in the past  four weeks)  3 = Often (more than ten times in the past four  weeks) |
|  | In the past four weeks, did you have to eat some foods that you really did not want to eat because of a lack of resources to obtain other types of food? | 0 = No (skip to Q5)  1 = Yes  ….\|___\| |
| 4.a. | How often did this happen? | 1 = Rarely (once or twice in the past four  weeks)  2 = Sometimes (three to ten times in the past  four weeks)  3 = Often (more than ten times in the past four  weeks)  ….\|___\| |
|  | In the past four weeks, did you have to eat a smaller meal than you felt you needed because there was not enough food? | 0 = No (skip to Q6)  1 = Yes  ….\|___\| |
| 5.a. | How often did this happen? | 1 = Rarely (once or twice in the past four  weeks)  2 = Sometimes (three to ten times in the past  four weeks)  3 = Often (more than ten times in the past four  weeks)  ….\|___\| |
|  | In the past four weeks, did you have to eat fewer meals in a day because there was not enough food? | 0 = No (skip to Q7)  1 = Yes  ….\|___\| |
| 6.a. | How often did this happen? | 1 = Rarely (once or twice in the past four  weeks)  2 = Sometimes (three to ten times in the past  four weeks)  3 = Often (more than ten times in the past four  weeks)  ….\|___\| |
|  | In the past four weeks, was there ever no food to eat of any kind for you because of lack of resources to get food? | 0 = No (skip to Q8)  1 = Yes  ….\|___\| |
| 7.a. | How often did this happen? | 1 = Rarely (once or twice in the past four  weeks)  2 = Sometimes (three to ten times in the past  four weeks)  3 = Often (more than ten times in the past four  weeks)  ….\|___\| |
|  | In the past four weeks, did you go to sleep at night hungry because there was not enough food? | 0 = No (skip to Q9)  1 = Yes  ….\|___\| |
| 8.a. | How often did this happen? | 1 = Rarely (once or twice in the past four  weeks)  2 = Sometimes (three to ten times in the past  four weeks)  3 = Often (more than ten times in the past four  weeks)  ….\|___\| |
|  | In the past four weeks, did you go a whole day and night without eating anything because there was not enough food? | 0 = No (the questionnaire is finished)  1 = Yes  ….\|___\| |
| 9.a. | How often did this happen? | 1 = Rarely (once or twice in the past four  weeks)  2 = Sometimes (three to ten times in the past  four weeks)  3 = Often (more than ten times in the past four  weeks)  ….\|___\| |

**Household questionnaire for assessment of food insecurity**

| S.No | Questions | Responses |
| --- | --- | --- |
|  | In the past four weeks, did you worry that your household would not have enough food? | 0 = No (skip to Q2)  1=Yes  ….\|___\| |
| 1.a. | How often did this happen? | 1 = Rarely (once or twice in the past four weeks)  2 = Sometimes (three to ten times in the past  four weeks)  3 = Often (more than ten times in the past four  weeks)  ….\|___\| |
|  | In the past four weeks, were you or any household member not able to eat the kinds of foods you preferred because of a lack of resources? | 0 = No (skip to Q3)  1=Yes ….\|___\| |
| 2.a. | How often did this happen? | 1 = Rarely (once or twice in the past four  weeks)  2 = Sometimes (three to ten times in the past  four weeks)  3 = Often (more than ten times in the past four  weeks)  \|___\| |
|  | In the past four weeks, did you or any household member have to eat a limited variety of foods due to a lack of resources? | 0 = No (skip to Q4)  1 = Yes  ….\|___\| |
| 3.a. | How often did this happen? | 1 = Rarely (once or twice in the past four  weeks)  2 = Sometimes (three to ten times in the past  four weeks)  3 = Often (more than ten times in the past four  weeks)  \|___\| |
|  | In the past four weeks, did you or any household member have to eat some foods that you really did not want to eat because of a lack of resources to obtain other types of food? | 0 = No (skip to Q5)  1 = Yes  ….\|___\| |
| 4.a. | How often did this happen? | 1 = Rarely (once or twice in the past four  weeks)  2 = Sometimes (three to ten times in the past  four weeks)  3 = Often (more than ten times in the past four  weeks)  ….\|___\| |
|  | In the past four weeks, did you or any household member have to eat a smaller meal than you felt you needed because there was not enough food? | 0 = No (skip to Q6)  1 = Yes  ….\|___\| |
| 5.a. | How often did this happen? | 1 = Rarely (once or twice in the past four  weeks)  2 = Sometimes (three to ten times in the past  four weeks)  3 = Often (more than ten times in the past four  weeks)  ….\|___\| |
|  | In the past four weeks, did you or any other household member have to eat fewer meals in a day because there was not enough food? | 0 = No (skip to Q7)  1 = Yes  ….\|___\| |
| 6.a. | How often did this happen? | 1 = Rarely (once or twice in the past four  weeks)  2 = Sometimes (three to ten times in the past  four weeks)  3 = Often (more than ten times in the past four  weeks)  ….\|___\| |
|  | In the past four weeks, was there ever no food to eat of any kind in your household because of lack of resources to get food? | 0 = No (skip to Q8)  1 = Yes  ….\|___\| |
| 7.a. | How often did this happen? | 1 = Rarely (once or twice in the past four  weeks)  2 = Sometimes (three to ten times in the past  four weeks)  3 = Often (more than ten times in the past four  weeks)  ….\|___\| |
|  | In the past four weeks, did you or any household member go to sleep at night hungry because there was not enough food? | 0 = No (skip to Q9)  1 = Yes  ….\|___\| |
| 8.a. | How often did this happen? | 1 = Rarely (once or twice in the past four  weeks)  2 = Sometimes (three to ten times in the past  four weeks)  3 = Often (more than ten times in the past four  weeks)  ….\|___\| |
|  | In the past four weeks, did you or any household member go a whole day and night without eating anything because there was not enough food? | 0 = No (the questionnaire is finished)  1 = Yes  ….\|___\| |
| 9.a. | How often did this happen? | 1 = Rarely (once or twice in the past four  weeks)  2 = Sometimes (three to ten times in the past  four weeks)  3 = Often (more than ten times in the past four  weeks)  ….\|___\| |

**Appendix B**

Interactions

| **Interaction terms** | **p-value in model** |
| --- | --- |
| Household FI*father schooling years | 0.97 |
| Household FI*mother schooling years | 0.59 |
| Mother schooling years*father schooling years | 0.66 |
| father schooling years*SES (1)  father schooling years*SES (2) | 0.22,  0.50 |

*p-value of < 0.20 was considered significant*
